# [Retracted] (‑)‑β‑hydrastine suppresses the proliferation and invasion of human lung adenocarcinoma cells by inhibiting PAK4 kinase activity

**DOI:** 10.3892/or.2025.8892

**Published:** 2025-04-01

**Authors:** Bingyu Guo, Xiaodong Li, Shuai Song, Meng Chen, Maosheng Cheng, Dongmei Zhao, Feng Li

Oncol Rep 35: 2246–2256, 2016; DOI: 10.3892/or.2016.4594

Following the publication of this paper, it was drawn to the Editor's attention by a concerned reader that certain of the flow cytometric data shown in Fig. 4A and Transwell cell migration and invasion assay data in Fig. 6A subsequently appeared in other published articles written by different authors at different research institutes (a pair of which have since been retracted owing to the striking similarity of these data with that featured in previously published articles). Moreover, overlapping data panels were also identified in Fig. 6A, such that these data, which were intended to show the results from differently performed experiments, had apparently been derived from the same original source.

Owing to the fact that the contentious data in the above article had apparently subsequently appeared in other unrelated articles, and given the fact that Fig. 6A had clearly been assembled incorrectly, the Editor of *Oncology Reports* has decided that this paper should be retracted from the Journal on the grounds of a lack of overall confidence in the originality of all the data presented therein. The authors were asked for an explanation to account for these concerns, but the Editorial Office did not receive a reply. The Editor apologizes to the readership for any inconvenience caused.

